# Does a monoclonal antibody targeting immune cells affect glutamatergic level in schizophrenia? A multimodal PET/MRS brain imaging study

**DOI:** 10.1038/s41386-026-02466-3

**Published:** 2026-07-09

**Authors:** Guy Hindley, Yuya Mizuno, Katherine Beck, Ines Carreira Figueiredo, Toby Pillinger, Sami Jeljeli, Joel Dunn, Alexander Hammers, Ramla Awais, Kerstin Sander, Erik Årstad, David J. Lythgoe, Julia J. Schubert, Mattia Veronese, Federico Turkheimer, Oliver D. Howes, Tiago Reis Marques

**Affiliations:** 1 Centre for Precision Psychiatry, Institute of Clinical Medicine, University of Oslo and Division of Mental Health and Addiction, Oslo University Hospital, 0407 Oslo, Norway; 2 Department of Psychiatry, Lovisenberg Diaconal Hospital, Oslo, Norway; 3 Department of Psychosis Studies, Institute of Psychiatry, Psychology and Neurosciences, King’s College London, 16 De Crespigny Park, London, SE5 8AB UK; 4 East London NHS Foundation Trust, London, UK; 5 Department of Neuropsychiatry, Keio University School of Medicine, Tokyo, Japan; 6 South London and Maudsley NHS Foundation Trust, London, UK; 7 School of Biomedical Engineering and Imaging Sciences, King’s College London, London, UK; 8 Centre for Radiopharmaceutical Chemistry, University College London, London, UK; 9 Department of Neuroimaging, Institute of Psychiatry, Psychology and Neurosciences, King’s College London, London, UK; 10 Department of Information Engineering, University of Padua, Padua, Italy

**Keywords:** Neuroimmunology, Schizophrenia

## Abstract

Microglial dysfunction and glutamatergic dysregulation are implicated in the neurobiology of schizophrenia. Microglia regulate brain glutamate levels through mechanisms including the cysteine-glutamate antiporter, raising the possibility that microglial dysfunction could underlie glutamatergic dysregulation in schizophrenia. We tested this using a combined cross-sectional/longitudinal study of individuals with first episode psychosis and healthy controls. We investigated two hypotheses: (1) increase in a positron emission tomography (PET) imaging marker of microglia is associated with increased anterior cingulate (ACC) glutamate levels; and (2) reducing immune trafficking into the CNS using the monoclonal antibody natalizumab would reduce ACC glutamate levels in people with first-episode psychosis. A total of 108 participants (68 patients and 40 healthy controls) underwent simultaneous proton magnetic resonance spectroscopy to quantify ACC glutamate and glutamate/glutamine (glx) levels and PET imaging with [18 F]DPA-714 to quantify translocator protein (TSPO) levels, a protein highly expressed by microglia in neuroinflammatory conditions. In the longitudinal arm, 50 first-episode psychosis patients were included and either randomised to receive natalizumab or placebo double-blind, or received open-label natalizumab. At baseline, there was no significant association between ACC glutamate concentration and ACC TSPO binding in the entire sample (β = -0.003, SE = 0.006, *p* = 0.55). In the longitudinal arm, natalizumab did not significantly alter ACC glutamate levels (mean difference = 0.227 i.u., t = 1.52, *p* = 0.139). These findings do not support the hypothesis that microglial dysfunction drives ACC glutamatergic dysregulation in early psychosis. Microglial dysfunction and glutamatergic dysregulation may therefore represent distinct mechanisms within the neurobiology of schizophrenia.

## Introduction

Schizophrenia is a psychotic disorder affecting approximately 1 in 100 people across their lifespan [1–3]. It ranks within the top 20 disorders for years lived with disability worldwide [4]. Current treatment options consist predominantly of dopamine D2 receptor blockers which are ineffective for one in three patients [5], have limited effects on negative and cognitive symptoms of schizophrenia [5], and are associated with cardiometabolic and other potentially detrimental side-effects [6]. An improved mechanistic understanding of the neurobiology of schizophrenia is required to develop novel treatment strategies and improve outcomes [7].

Several lines of evidence support a role for microglial dysfunction in the neurobiology of schizophrenia [8]. Post-mortem studies have shown increased microglial density and increased translocator protein (TSPO) expression, a protein known to be highly expressed by microglia in neuroinflammatory conditions [9] and in the brains of individuals with schizophrenia [10, 11]. Some positron emission tomography (PET) studies have also shown elevated TSPO binding in total grey matter (GM) in people with schizophrenia compared to healthy controls, particularly when using a reference region approach [12, 13]. Nevertheless, findings are mixed, with one meta-analysis of in vivo TSPO PET studies finding lower TSPO levels [13]. Discrepancies are likely attributable to differences in radioligand properties, quantification approaches (e.g., reference region versus arterial input function methods), reduced tracer permeability in schizophrenia [14], clinical stage, and patient heterogeneity, reflecting the broader ongoing debate regarding the role of neuroinflammation in schizophrenia [15].

Dysregulation of glutamate, the brain’s primary excitatory neurotransmitter, is also implicated in the neurobiology of schizophrenia [16]. Supporting evidence includes human post-mortem findings showing reduced expression of glutamate receptor subunits in multiple brain regions, including the anterior cingulate cortex (ACC) [17–19]. Concentrations of glutamate and its metabolite glutamine can be estimated in vivo using proton magnetic resonance spectroscopy (^1^H-MRS) [20]. ^1^H-MRS studies in schizophrenia have found that glutamate levels are positively associated with greater symptom severity in the medial frontal cortex [21] and negative and cognitive symptoms specifically in the ACC in treatment-resistant schizophrenia [22]. Furthermore, higher ACC glutamate and glutamine concentrations (glx) at the beginning of treatment was associated with poorer response to antipsychotic treatment [23, 24].

Interestingly, microglia are known to regulate synaptic glutamate levels via several pathways [25–27]. Likely contributing to glutamate homoeostasis under physiological conditions, microglial activation can result in substantial increases in extracellular glutamate levels directly via increased activity of the cystine-glutamate antiporter [27] and indirectly via increases in TNF-α release, which blocks astrocytic glutamate reuptake and increases astrocytic glutamate [28]. Critically, both mechanisms act by increasing the total extracellular glutamate pool, which would be expected to elevate glutamate levels as detectable by ¹H-MRS (which measures the total glutamate pool in the region of interest). This suggests that microglial dysfunction could underlie glutamatergic dysregulation associated with schizophrenia as measured by ^1^H-MRS. So far, the association between microglial dysfunction and glutamate dysregulation in first-episode psychosis has been investigated in a single study, showing no significant association between PET TSPO levels and hippocampal glutamate concentration, as measured by ^1^H-MRS [29]. However, the study did not include other brain regions of interest such as the ACC, and, to our knowledge, no study has tested the effect of an immune modulator on brain glutamate levels.

In view of this, we explored for the first time the relationship between TSPO binding and ACC glutamate and glx in symptomatic individuals with first-episode psychosis and healthy controls. Prior to initiation of the study, we hypothesised that ACC TSPO levels would be positively associated with glutamate levels both in patients and controls. Our second hypothesis was that pharmacological reduction of PET TSPO levels in the ACC using natalizumab would be associated with reductions in ACC glutamate and glx levels in patients. We included both glutamate and glx given previous positive findings in the ACC for both measures, as described above [21–24]. We used natalizumab based on evidence of robust reductions in PET TSPO levels following 3-month treatment in patients with multiple sclerosis [30].

## Methods

The Inflammatory Response in Schizophrenia (IRIS) study received ethical approval from the London - West London & GTAC Research Ethics Committee (reference: 17/LO/0002). The study was preregistered at ClinicalTrials.gov (NCT03093064). Written informed consent was provided by all participants prior to participation in accordance with the Declaration of Helsinki.

### Design

The study used a cross-sectional, case-control design followed by a nested longitudinal design to evaluate the effects of natalizumab in the patient group only [31]. There were two phases to the longitudinal evaluation. The first phase comprised a placebo group as a comparator to the effects of natalizumab in the active group, employed within a double-blind, randomised, parallel-group design, with a 1:1 patient allocation to either the natalizumab or placebo group. The second phase comprised the administration of open-label natalizumab, initiated due to disruption to the study caused by the coronavirus pandemic. In both phases, treatment with natalizumab comprised a total of three infusions of 300 mg natalizumab once a month, if tolerated. The placebo group in the first phase received NaCl 0.9% saline infusions of matching volume.

### Sample size calculation

Sample size was defined according to the primary outcome of this study, total grey matter TSPO binding, as reported in [31]. For the cross-sectional analysis, target sample sizes were based on findings by Van Berkel and colleagues who demonstrated enhanced [11 C]-PK11195 binding potential in individuals with early onset schizophrenia compared with controls with an effect size of d = 0.87 [32]. Using G*POWER, a sample size of 22 in each group (total 44) would provide >80% power to detect this effect size using a paired t-test for alpha = 0.05 (two-tailed). For the longitudinal analysis, target sample sizes were based on a power calculation which gave >80% power to detect a 0.83 effect size difference in mean change between the two groups with 24 patients in each group. Based on previous longitudinal PET studies we expected a dropout of <15%. However, to be conservative we allowed for an attrition rate of 20% between the baseline and follow-up PET. We therefore aimed to recruit up to 30 schizophrenia patients per group at baseline.

### Sample

Participants were recruited to the study between October 2017 and June 2023. Cases were defined as individuals with first-episode psychosis who fulfilled the following inclusion criteria: (1) met DSM-5 diagnostic criteria for schizophrenia or other psychotic disorder; (2) 18–50 years old; (3) less than 5 years duration of illness; (4) symptomatic despite ongoing treatment with antipsychotic medications, defined as at least one positive symptom and at least one negative symptom rated as a three or more on the Positive and Negative Syndrome Scale (PANSS); and (5) taking a stable dose of any antipsychotic medication besides clozapine for a minimum of one month before screening.

Healthy controls were defined as 18-50 year-old individuals with no current or previous psychiatric disorder determined using SCID-5 Clinical Version [33, 34], and no first-degree relatives with schizophrenia or other psychotic disorder.

Exclusion criteria for both cases and controls included: (1) a history of co-morbid CNS disorder, head trauma, significant infection, immunocompromised state, auto-immune or inflammatory condition, or other serious acute or chronic illness which the investigators thought might affect study measures or safety, e.g. by affecting immune system function; (2) infection within the preceding 3 months; (3) ongoing and long-standing use of oral steroids or non-steroidal anti-inflammatory drugs; (4) substance use or dependence besides nicotine; (5) current pregnancy or breast-feeding; (6) previous use of monoclonal antibodies including natalizumab; (7) clinical findings after physical examination, routine screening blood tests or ECG deemed significant by the study investigators; (8) low affinity binder status to TSPO as determined by TSPO genotyping, performed using a TaqMan genotyping assay which amplifies the genomic region using real time PCR before testing for the wild type and mutant alleles using specific probes [35]; (9) contraindications to MRI. Cases were additionally excluded for: (9) contraindications to natalizumab and (10) enrolment within the last 30 days to a clinical study of unlicensed medication.

Cases were recruited from Early Intervention Psychosis services within four London mental health trusts. Controls were recruited from the equivalent London area through online advertisement.

### Demographic and clinical measures

During an initial screening interview, participants provided basic demographic information including age, sex and ethnicity. All participants underwent the Structured Clinical Interview for DSM (SCID-5-CV), administered by a psychiatrist, to either confirm or exclude a diagnosis of schizophrenia, other psychotic disorder or other co-morbid psychiatric disorder [33]. Cases were evaluated for current symptom burden using the PANSS [36], Scale for the Assessment of Negative Symptoms (SANS) [37], and the Calgary Depression Scale for Schizophrenia (CDSS) [38]. Cases provided a history of antipsychotic use, which was converted to chlorpromazine equivalent exposure and daily dose [39].

### Data acquisition

Participants received a bolus injection of [^18^F]DPA-714 immediately before undergoing a dynamic, continuous, 60-min PET-MR scan acquired by a 3-Tesla Siemens Biograph PET-MR scanner (Siemens Healthcare, Erlangen, Germany).

For ^1^H-MRS acquisition, a sagittal 3D T1-weighted structural magnetisation-prepared rapid gradient echo (MPRAGE) MRI scan was acquired prior to voxel prescription (see Supplementary Fig. 1). The ^1^H-MRS voxel, which measured 2 cm × 2 cm × 2 cm (i.e.8 cm^3^), was positioned in the midline, with the centre of the voxel 16 mm superior to the anterior portion of the genu of corpus callosum. ^1^H-MRS spectra were subsequently acquired from the ACC, using point resolved spectroscopy (PRESS) pulse sequence with an echo time of 30 msec, repetition time of 3000 msec, 2048 acquisition points, an acquisition bandwidth of 2500 Hz and 128 averages. Water signal suppression was performed using Chemically Selective Suppression (CHESS). Water unsuppressed spectra were acquired separately with 16 averages. Vendor automated adjustments were used to optimise shimming and water suppression prior to MRS spectra acquisition. Spectra were also manually shimmed to ensure a full width at half maximum less than or equal to 20 Hz. An example spectrum is provided in Supplementary Fig. 2.

PET reconstruction was performed dynamically in 26 frames: 1×60 sec, 8×15 sec, 3×1 min, 5×2 min, 9×5 min. Simultaneous T1-weighted structural MRI data were collected for co-registration. All participants additionally underwent a low-dose head CT scan (140 kV, 10 mA, helical acquisition) after completion of the PET-MR for tissue attenuation correction of the PET image [40]. The CT was acquired using a GE Discovery DST 710 PET/CT (GE Healthcare, Chicago, Illinois, USA).

### ^1^H-MRS analysis

We used LC-model version 6.3-1 L to quantify water-scaled glutamate and glx concentrations with eddy current correction [41]. Spectra were excluded if the following quality control criteria were not met: (1) Cramer-Rao lower bounds (CRLB) for all metabolites less than or equal to 20%; (2) signal-to-noise ratio greater than or equal to 10; and (3) line-width (full-width at half-maximum) greater than or equal to 0.1 ppm. Metabolite concentrations were corrected for cerebrospinal fluid (CSF) content by segmenting the voxel into white matter (WM), GM, and CSF using SPM 12 and Gannet 3.1 (Supplementary Fig. 1). Metabolite concentrations were then corrected using the following formula [42], where M = metabolite concentration and f = fraction of:

$${Mcorr}={Mraw} ^{*} \left(1.207^{*} {fGM}+{fWM}+1.548^{*} {fCSF}\right)/(1-{fCSF})$$

Metabolite concentrations are reported in institutional units (i.u.), which means that the measures reported are dependent on local acquisition specifics such that they cannot be compared across studies from other sites [43]. See Supplementary Table 1 for comparison of quality control values between groups.

### PET analysis

PET pre-processing was performed using MIAKAT (version 4.2.6) [44], a PET analysis toolbox package built in MATLAB (version 2015b; The MathWorks, Inc., Natick, MA, USA). Brain extraction and tissue segmentation were performed on MPRAGE MRI data, which were then linearly aligned to standard MNI space. The Clinical Imaging Centre (CIC) neuroanatomical atlas v2.0 was then non-linearly transformed into each participant’s MPRAGE space using the MNI-to-subject deformation field and combined with individual GM segmentations to define GM regions of interest (ROIs). The ACC served as the primary ROI; total GM, frontal GM, and temporal GM were included as exploratory ROIs [44, 45].

For PET data, frame-by-frame motion-correction was applied to the dynamic series, which were then co-registered with the MPRAGE MRI and the transformed CIC atlas. Time activity curves (TACs) were then extracted from each ROI. All pre-processed MRI and PET data underwent manual quality control by researchers blinded to treatment condition to ensure usability of the data. PET data was investigated for motion artefact, poor signal to noise ratio, and, after processing, for inappropriate anatomical alignment between images and the anatomical atlas. See Supplementary Fig. 3 for examples of extracted PET data and their co-registered ROIs.

TSPO is ubiquitously expressed in the brain, and therefore no true reference region exists for quantification. We therefore applied a pseudo-reference region approach that has been previously validated for TSPO tracers [46, 47]. Briefly, GM voxels exhibiting kinetic behaviour most consistent with minimal specific binding, similar to that expected in healthy GM, were identified using a supervised clustering algorithm [48]. Distribution volume ratios (DVR) were then estimated for each ROI using a simplified reference tissue model that includes correction for tracer binding to vascular tissue [49]. See Supplementary Figs. 4, 5 for further details of the pseudo-reference region approach.

### Statistical analysis

Data were analysed using the Tidyverse package v2.0.0 in R v3.6.0 operated through RStudio v1.3.1093. Figures were generated using the ggplot2 package, v3.4.2. The distribution of continuous variables was investigated by visual inspection of QQ plots and formally tested using the Shapiro-Wilk test. Homogeneity of variances between patient and control groups was determined using the F-test. Demographic differences between cases and controls were tested for using Student’s t-test since all variables were normally distributed, and chi-squared test for categorical variables besides those with one or more cell with an expected count less than five, for which Fisher’s exact test was used.

Group differences in ACC glutamate and glx between patients and controls were tested for using two-sided Student’s t-test. For ACC TSPO binding, for which TSPO genotype and age are known to effect TSPO binding [50], analysis of covariance (ANCOVA) was used to test the effects of group on the dependent variable ACC TSPO DVR with age and TSPO genotype (i.e. high affinity or mixed affinity binder status) included as covariates.

In order to test our main hypotheses, the association between ACC TSPO DVR and ACC glutamate and glx in the whole sample was calculated using a linear regression model with ACC glutamate or glx as the dependent variable. Since TSPO binding is affected by age, TSPO genotype, and smoking status and ACC glutamate and glx are affected by age, sex, and smoking status, we performed four additional sensitivity analyses: (1) a model controlling for TSPO binding status only, (2) a model controlling for TSPO binding status, age, and sex; (3) the simplified model within high affinity binders only, and (4) a model controlling for smoking status only. We subsequently tested for associations between ACC glutamate and glx and ACC TSPO DVR in patients and controls separately in an exploratory analysis. We performed additional exploratory analyses testing for associations between total, frontal lobe, and temporal lobe GM TSPO DVR and ACC glutamate and glx. The results of the total, frontal and hippocampal, but not ACC, PET analysis have been previously reported [31]. None of the other data or analyses have been published before.

We also tested for the association between ACC metabolite concentration and symptom severity using linear regression models, with symptom score used as the dependent variable, and age and sex included as covariates. We included total PANSS score, positive, negative, and general symptom subs-scale scores, SANS total score and CDSS total score (supplementary results, Supplementary Fig. 6).

In the longitudinal arm of the study, we analysed pooled data from patients with first-episode psychosis who received either randomised or open-label infusions of natalizumab to test for evidence of an effect of natalizumab treatment. This approach was taken because of the combined study design which incorporated both an RCT phase and an open-label phase. We first tested for a change in ACC glutamate and glx concentrations in all patients receiving natalizumab using paired t-tests since a negative result was sufficient to reject our hypothesis despite the heterogeneity introduced by including two study designs. If there was a significant reduction in ACC glutamate or glx concentrations within the treatment group, we planned to subsequently test for a difference in effect between treatment and control groups. We performed an additional sensitivity analysis within participants who received all three natalizumab infusions. To investigate the effects of covariates, we also tested for an effect of time on ACC glutamate and glx concentrations after controlling for age,sex and smoking status using ANCOVA.

Since we hypothesised that natalizumab would cause reductions in ACC glutamate and glx levels via reduction of TSPO levels, we also tested whether baseline ACC TSPO DVR predicted change in ACC glutamate and glx levels using linear regression. We performed additional sensitivity analyses including age, sex and TSPO genotype as covariates and restricting the sample to participants who received all three doses of natalizumab. Since there is no evidence that increased TSPO binding in patients is specific to the ACC, we performed additional exploratory analyses testing whether total, frontal lobe, or temporal lobe GM TSPO DVR predicted change in ACC glutamate or glx. As there was no evidence of an effect of natalizumab on total GM or temporal lobe GM TSPO DVR in the primary study [31], we performed an additional exploratory analysis testing for an association between change in ACC TSPO DVR and change in ACC glutamate and glx in the entire first-episode psychosis sample using linear regression. We performed sensitivity analyses including TSPO genotype, age and sex as covariates in the model, and restricting the sample to participants who received all three infusions. We also tested for associations between change in total, frontal lobe, and temporal lobe GM TSPO DVR and change in ACC glutamate or glx.

Since there was a reported reduction in PANSS total, positive and negative symptoms scores within the treatment group [31], we used linear regression to test whether changes in ACC glutamate and glx concentration were associated with change in symptoms in response to natalizumab. Change in PANSS scores were calculated as percentage change after subtracting minimal values (e.g. 30 for PANSS total scores) to convert the scale into a ratio scale [51].

All analyses were carried out with a two-tailed p-value of 0.05. For analyses testing the association between ACC TSPO DVR and ACC glutamate and glx in both cross-sectional and longitudinal arms, we did not correct for multiple comparisons as these outcomes were defined as primary. For other analyses, we adjusted *p* values using Bonferroni correction. Glutamate and glx were not adjusted for multiple testing as they were defined as primary outcomes for the study.

## Results

### Sample

A total of 74 patients and 42 healthy controls were recruited and completed baseline assessments. After quality control (see Supplementary Results for further details), 68 patients and 40 controls had useable MRS data, of which 61 patients and 39 controls also had useable PET data at baseline. Demographics and clinical features of the full MRS sample are presented in Table 1. There were no statistical differences between patients and controls with regards to age, sex, ethnicity or TSPO genotype status.

**Table 1:** Table summarising sample make-up, demographics, and clinical features of the full MRS sample.

	Patients with first episode psychosis			Healthy Controls	Statistical test
	*Randomised natalizumab*	*Open label*	*Placebo*		Patients vs. controls in MRS sample
**n MRS (n PET + MRS)**	68 (61)			40 (39)	
	*17 (15)*	*15 (14)*	*18 (16)*		
**Age - mean (SD)**	27.5 (7.53)			28.3 (7.86)	t = -0.532, p = 0.596
	*26.9 (8.98)*	*27.9 (5.62)*	*28.3 (8.32)*		
**Sex - n female (%)**	24 (35.3)			18 (45.0)	χ^2^ = 0.632, p = 0.427
	*9 (52.9)*	*9 (60.0)*	*11 (38.9)*		
**Ethnicity – n (%):**					
White	13 (19.1)			14 (35.0)	p = 0.138
	*3 (17.6)*	*4 (26.7)*	*4 (22.2)*		
Black African/Caribbean*	34 (50.0)			11 (27.5)	
	*9 (52.9)*	*6 (40.0)*	*10 (55.6)*		
Asian	11 (16.2)			9 (22.5)	
	*3 (17.6)*	*3 (20.0)*	*1 (5.56)*		
Mixed ancestry	8 (11.8)			4 (10.0)	
	*1 (5.88)*	*2 (13.3)*	*2 (11.1)*		
Other	2 (2.94)			2 (5.00)	
	*1 (5.88)*	*0 (0)*	*1 (5.56)*		
**Diagnosis – n (%):**					
Schizophrenia	44 (64.7)			N/a	
	*9 (52.9)*	*11 (73.3)*	*13 (72.2)*		
Schizophreniform disorder	14 (20.6)			N/a	
	*4 (23.5)*	*4 (26.7)*	*3 (16.7)*		
Unsp schizophrenia spect. other psychotic disorder	6 (8.82)			N/a	
	*2 (11.8)*	*0 (0)*	*2 (11.1)*		
Schizoaffective disorder	4 (5.88)			N/a	
	*2 (11.8)*	*0 (0)*	*0 (0)*		
**Medication status**					
Aripiprazole (2)	33 (48.5)				
	*10 (58.8)*	*6 (40.0)*	*8 (44.4)*		
Paliperidone/risperidone (10)	13 (19.1)				
	*2 (11.8)*	*4 (26.7)*	*4 (22.2)*		
Quetiapine (9)	4 (5.88)				
	*1 (5.88)*	*0 (0)*	*1 (5.56)*		
Olanzapine (8)	14 (20.6)				
	*3 (17.6)*	*3 (20.0)*	*4 (22.2)*		
Amisulpride (2 – 2)	2 (2.94)				
	*1 (5.89)*	*0 (0)*	*1 (5.56)*		
Haloperidol (6)	1 (1.47)				
	*0 (0)*	*1 (6.67)*	*0 (0)*		
Flupentixol (5)	1 (1.47)				
	*0 (0)*	*1 (6.67)*	*0 (0)*		
**TSPO genotype (PET sample only) - n HABs (%)**	40 (65.6)			29 (74.4)	χ ^2^ = 0.858, p = 0.354
	*7 (46.7)*	*11 (78.6)*	*11 (68.8)*		
**Total PANSS - mean (SD)**	57.3 (12.3)			N/a	
	*55.5 (10.5)*	*51.9 (11.0)*	*58.8 (13.6)*		
**CDSS total - mean (SD)**	2.75 (3.10)			N/a	
	*3.47 (3.62)*	*1.67 (2.61)*	*2.44 (2.33)*		
**SANS total - mean (SD)**	8.00 (3.44)			N/a	
	*7.65 (3.37)*	*6.6 (4.45)*	*8.56 (3.13)*		

For patients with first-episode psychosis, the cross-sectional MRS sample is described in the first row for each variable, and the longitudinal sample broken down into those randomised to natalizumab, receiving open label natalizumab and randomised to placebo are described in the second row for each variable. Differences between first episode psychosis patients and healthy controls were tested using Student t test (age), Chi-squared test (sex), and Fisher’s exact test (ethnicity). *n* frequency, *SD*standard deviation, *TSPO* translocator protein, *HAB* high affinity binder, *PANSS* positive and negative syndrome scale, *CDSS* Calgary depression scale for schizophrenia, *SANS* scale for the assessment of negative symptoms.

For the longitudinal phase of the study, 48 patients underwent randomisation to either natalizumab (*n* = 24) or placebo (*n* = 24). Eighteen additional patients received open-label natalizumab infusions. Of these 66 patients, 47 underwent follow-up PET-MR scans and four underwent follow-up MRI only due to late delivery of the PET tracer at baseline. The final dataset consisted of 32 participants who received natalizumab (17 randomised, two of whom underwent MRS only, and 15 open-label, one of whom underwent follow-up MRS only) and 18 randomised to placebo, two of whom underwent MRI only. See Supplementary Fig. 7 for the CONSORT flow diagram describing participant recruitment.

### Cross-sectional ACC glutamate and glx concentrations and ACC TSPO comparisons between the patient and control groups

ACC glutamate concentration, glx concentration and TSPO DVR were all normally distributed in both patients and controls. There were no significant differences in the variance of ACC metabolite concentrations or ACC TSPO DVR in patients and controls (F-test for homogeneity of variance *p* > 0.05). There was significantly lower ACC glx concentration in patients (mean = 11.1, s.d. = 1.09) compared to controls (mean = 11.6, s.d. = 0.819; t = -2.08, p = 0.040), but no significant group differences in ACC glutamate concentration (t = -1.45, p = 0.151) or ACC TSPO DVR (F = 1.67, *p* = 0.199) (Supplementary Fig. 8). As reported elsewhere, TSPO DVR was significantly higher in patients compared to controls in total (η^2^ = 0.043, *p* = 0.038) and temporal lobe (η^2^ = 0.057, *p* = 0.016) but not frontal lobe (η^2^ = 0.007, *p* = 0.406) GM [31].

### The relationship between TSPO DVR and glutamate and glx measures in patients and healthy controls

**Fig. 1:**
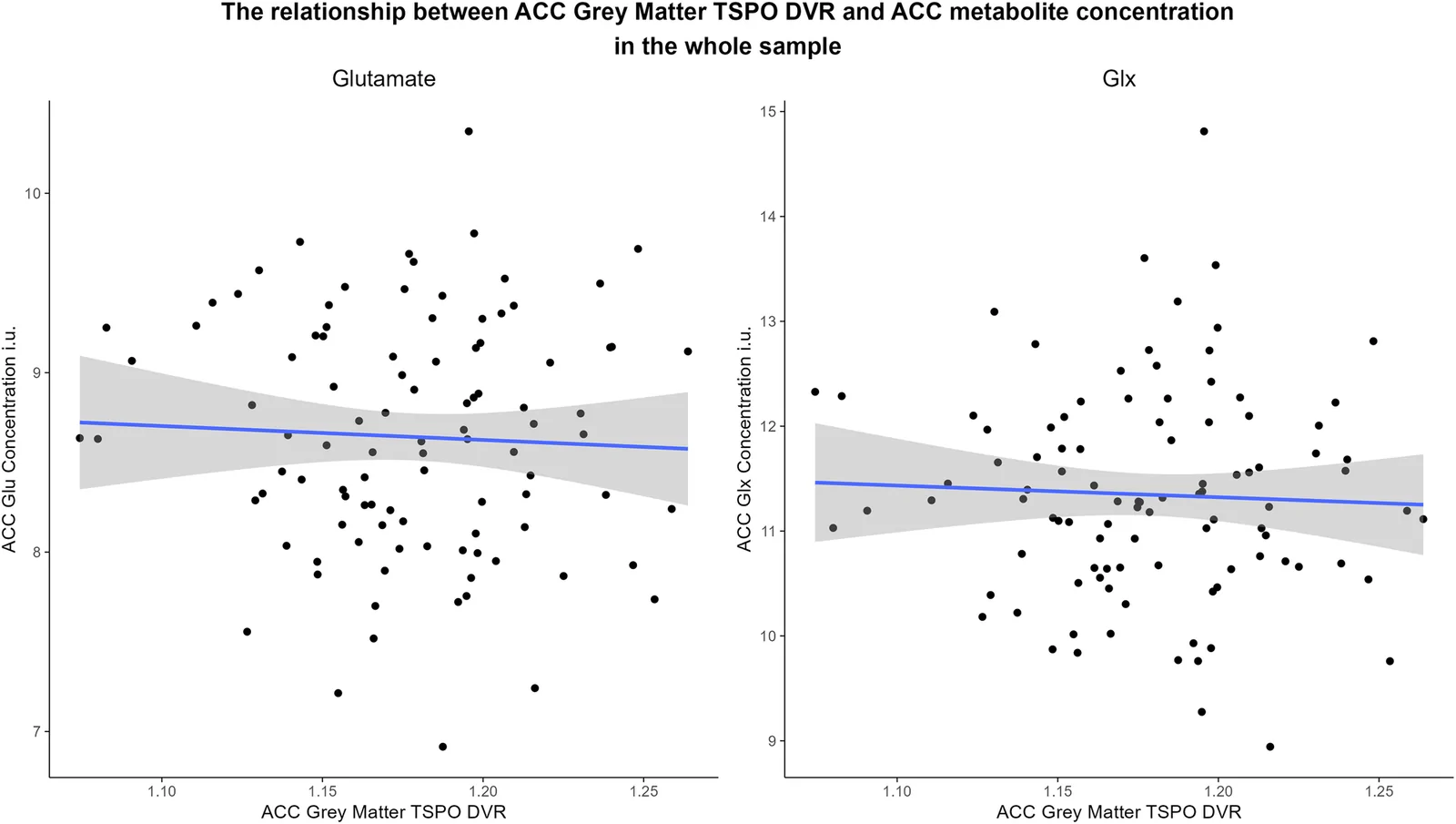
Scatter plots illustrating the relationship between ACC grey matter TSPO distribution volume ratios (DVR) (x axis) against ACC metabolite concentrations (y axis) in the whole sample. There was no significant relationship between any measure. Glu glutamate, glx glutamate + glutamine. Line of best fit and its standard errors are estimated using a linear model.

**Fig. 2:**
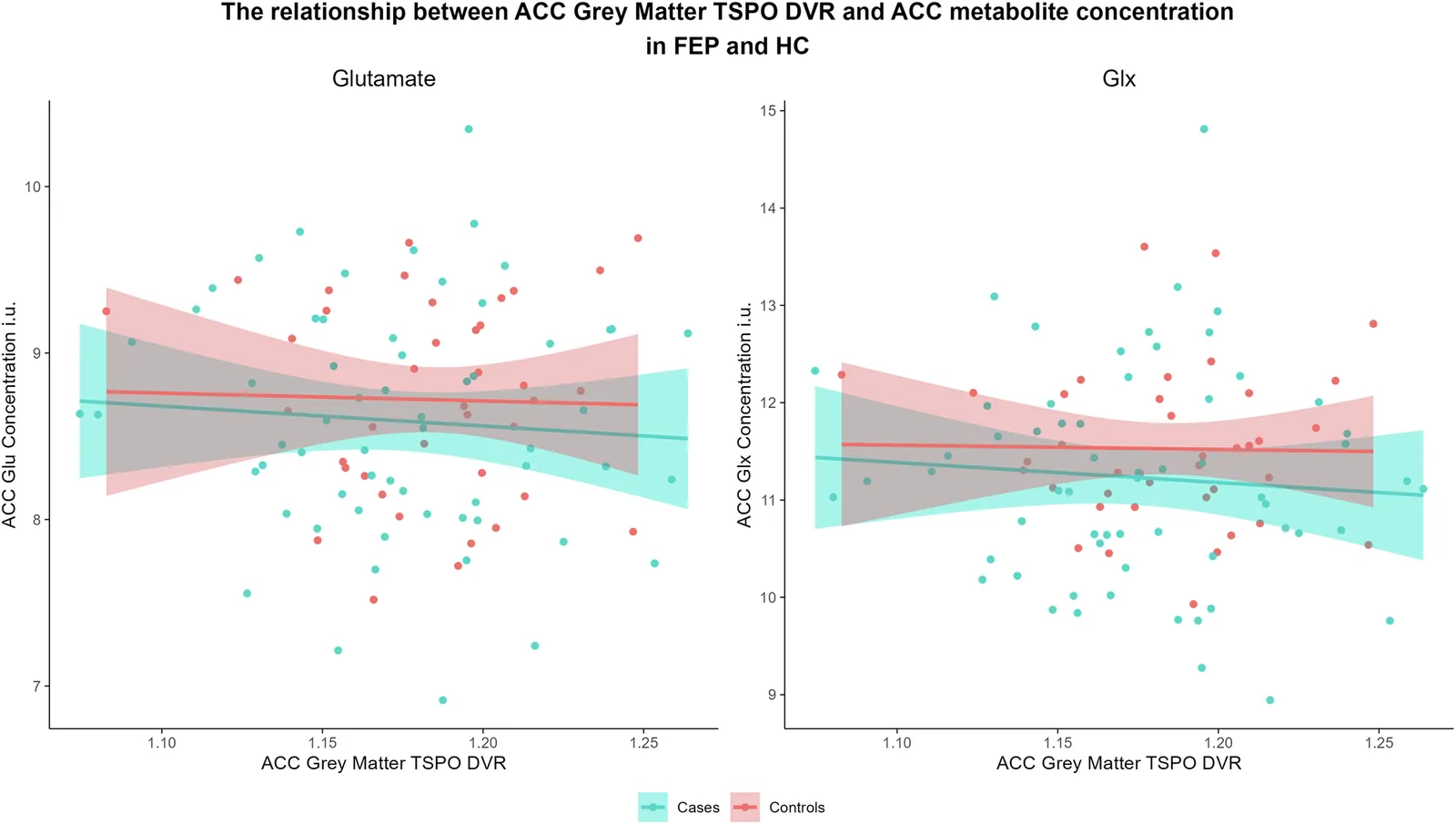
Scatter plots illustrating the relationship between ACC grey matter TSPO distribution volume ratios (DVR) (x axis) against ACC metabolite concentrations (y axis) in patients (blue) and controls (red). There were no significant associations between any of the measures. Glu glutamate, glx = glutamate + glutamine. Line of best fit and its standard errors are estimated using a linear model.

There were no significant associations between ACC TSPO DVR and ACC glutamate or glx in the whole sample (Fig. 1), or in patient or control groups individually (Fig. 2, Table 2). These findings remained non-significant when including only TSPO genotype as a covariate, TSPO genotype, age and sex as covariates, when restricting the sample to HABs and when controlling for current smoking status (Supplementary Tables 2–5). In exploratory analyses, there were no significant associations between TSPO DVR in other brain regions (total, frontal lobe or temporal lobe GM) and ACC glutamate or glx concentrations either in the whole sample, or in patients or controls individually (Supplementary Tables 6–8).

**Table 2:** Testing for an association between ACC TSPO DVR and ACC glutamate (glu) and glutamate + glutamine (glx) concentrations using linear regression in the whole sample, patients with first episode psychosis and healthy controls.

ACC metabolite	Whole sample (*n* = 100)	Patients with first episode psychosis (*n* = 61)	Healthy controls (*n* = 39)
Glu	β = -0.003, SE = 0.006, p = 0.553	β = -0.009, SE = 0.008, p = 0.244	β = 0.001, SE = 0.009, p = 0.865
Glx	β = -0.004, SE = 0.004, p = 0.263	β = -0.008, SE = 0.005, p = 0.117	β = -0.001, SE = 0.006, p = 0.908

There were no significant associations either for the whole sample or within patient or control groups.

### Change in glutamatergic measures in ACC following natalizumab treatment

**Fig. 3:**
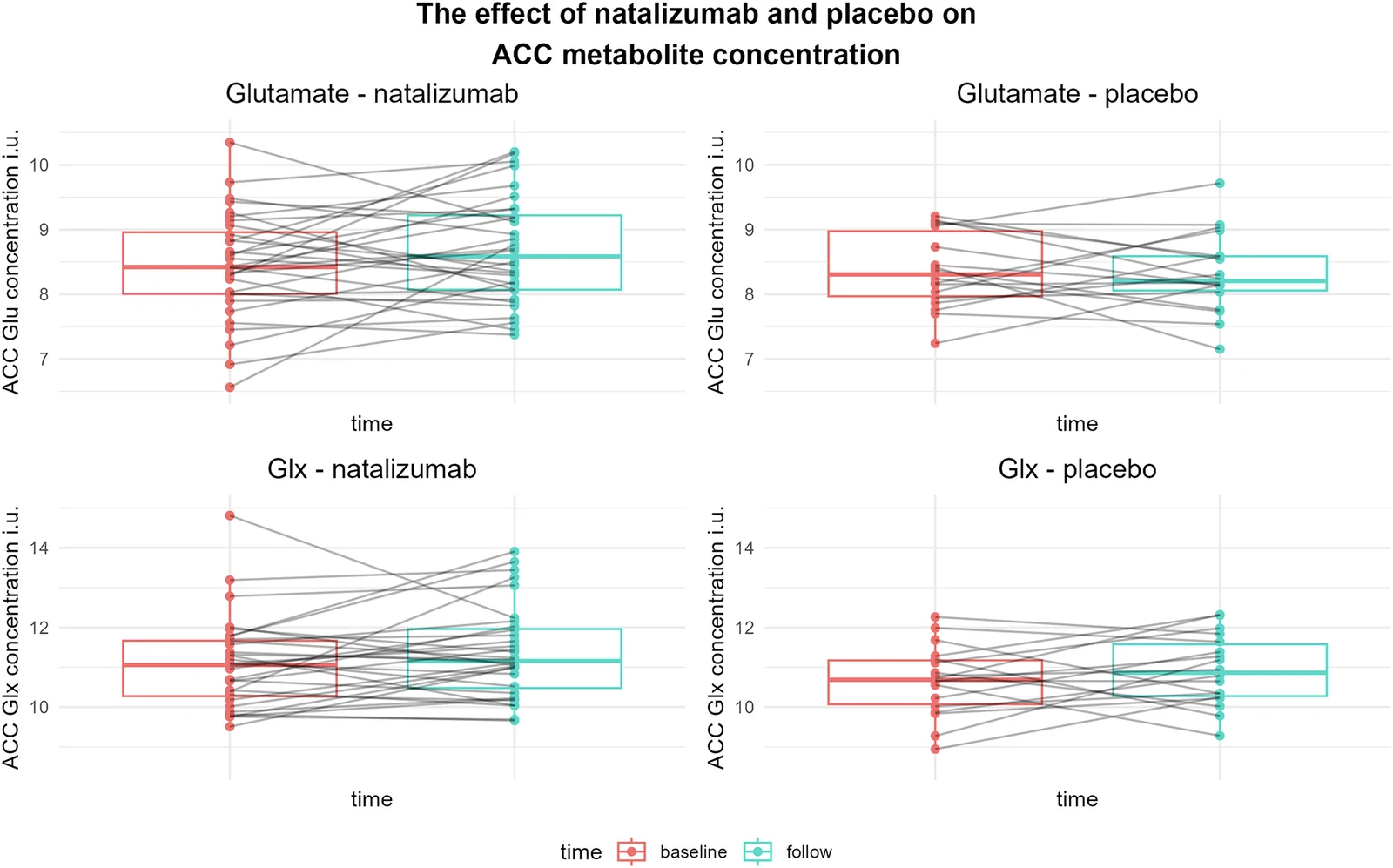
Box plots illustrating the effect of natalizumab and placebo infusions on ACC glutamate and glutamate + glutamine (glx) concentrations (y axis). There was no significant effect of natalizumab on glutamate or glx concentrations. Baseline data is presented in red and follow-up data is presented in blue.

There were no significant changes in either ACC glutamate concentration (mean difference = 0.227, *t* = 1.52, *p* = 0.139) or glx concentration (mean difference = 0.296, *t* = 1.59, *p* = 0.083) in patients who received natalizumab (Fig. 3). This remained non-significant when restricting the sample to individuals who received three natalizumab doses (glutamate: mean difference = 0.281, t = 1.73, p = 0.095; glx: mean difference = 0.391, t = 1.95, p = 0.062) and when controlling for age, sex and current smoking status (Supplementary Table 9). For comparison, mean change in ACC glutamate and glx concentrations among patients after receiving placebo were -0.0457 (t = -0.282, p = 0.781) and 0.257 (t = 1.06, *p* = 0.303), respectively (Fig. 3). Our study was, however, not powered to identify differences within the placebo group.

### Change ACC glutamate and glx and change in symptom measures after treatment with natalizumab

Since there was a significant change in PANSS total, positive and negative symptom scores after treatment with natalizumab [31], we tested whether change in PANSS scores was associated with change in glutamate or glx. There was no significant association between change in either ACC glutamate or glx concentrations and change in PANSS total score (glutamate: n = 32, β = -0.690, SE = 10.6, p = 0.948; glx: n = 32, β = -0.852, SE = 8.50, p = 0.921), percentage change in PANSS positive score (glutamate: n = 32, β = -1.49, SE = 12.8, p = 0.908; glx: n = 32, β = -9.52, SE = 10.1, p = 0.355), or percentage change in PANSS negative scores (glutamate: n = 32, β = -4.28, SE = 12.9, p = 0.742; glx: n = 32, β = 6.52, SE = 10.3, p = 0.532). These findings remained non-significant when controlling for age and sex (Supplementary Table 10) and when restricting the sample to individuals who received all three infusions (Supplementary Table 11).

## Discussion

To our knowledge, this is the first PET/MR study to explore the relationship between TSPO binding and ACC glutamate and glx in patients with first-episode psychosis and healthy controls, and the effect of natalizumab on ACC glutamate and glx. Contrary to our primary hypothesis, there was no significant association between higher TSPO DVR and higher glutamate or glx levels in the ACC in either patients or controls. Furthermore, the lack of significant associations within our patient sample alone suggests that this is unlikely to represent a disorder-specific mechanism.

Although these negative findings are in contrast to preclinical studies showing that microglial activation increases synaptic glutamate [25–28], they are supported by the only other clinical study investigating TSPO binding and brain glutamate levels as measured by ^1^H-MRS [29] which reports no association between hippocampal glutamate levels and TSPO total distribution volume (V_T_) in individuals at clinical high risk, patients with first-episode psychosis, healthy controls, or the entire sample combined.

There are several potential reasons for the lack of associations reported in the present study. First, TSPO is also expressed by other cell types including astrocytes and endothelial cells [9, 52]. The TSPO DVR both in patients and controls may therefore include other sources of TSPO expression, diluting relationships. Furthermore, a meta-analysis of post-mortem studies has shown that there are differences in microglial gene expression in individuals with schizophrenia compared to controls, suggestive of microglial dysfunction, but not evidence of increased microglial activation [53]. These findings introduce further uncertainty regarding the extent to which differences in TSPO expression in schizophrenia compared to controls is driven by microglial activation and should be considered in the context of our primary hypothesis. More specific markers of microglia, particularly those reflecting functional phenotypes, are required to test this further. Second, MRS and PET have limited spatial sensitivity, generating estimates of metabolite concentration and ligand binding averaged across a voxel or ROI that contains millions of individual synapses. It is therefore possible that microglial dysfunction alters glutamate at the level of the synapse, but this association is not observable using current MRS and PET methodologies [54]. Third, it is possible that there was insufficient variability in microglial activation within our sample to cause a measurable difference in ACC glutamate or glx. This could be expected in the control sample, which may not exhibit sufficient amounts of microglial activation to result in substantial differences in glutamate or glx concentrations. Nevertheless, we also found no evidence of an association in first-episode psychosis, despite previous findings showing increased relative TSPO PET tracer binding compared to controls [12]. There was, however, no significant difference in TSPO DVR in the present sample in the frontal lobe [31] or, as reported here, the ACC. We also unexpectedly found reduced glx levels in patients compared to controls, but no significant difference in glutamate levels. It is therefore possible that the present sample of first-episode psychosis patients did not exhibit sufficient microglial dysfunction in the ACC to result in a measurable difference in ACC glutamate or glx levels. This may be because the present sample experienced a relatively mild symptom burden, reflected by an average PANSS total scores at baseline of 57.3, equivalent to a “mildly ill” patient [55]. Differences in ACC glutamate and frontal lobe TSPO DVR may be more pronounced within a more symptomatic cohort.

Alternatively, the negative findings from the cross-sectional analysis may represent a true negative relationship between ACC glutamate dysregulation and increased TSPO expression. In addition to the previously reported lack of association between hippocampal glutamate levels and total TSPO DVR [29], this is supported by the fact that we identified differences in TSPO expression in total and temporal GM but not frontal GM [31], nor ACC GM specifically. Schizophrenia is a highly heterogenous disorder defined exclusively by clinical assessment [56]. It is therefore possible that schizophrenia has multiple distinct pathophysiological mechanisms which lead to overlapping clinical presentations [3]. Notwithstanding possible reasons for type II error described above, these findings suggest that TSPO overexpression and ACC glutamatergic dysregulation are distinct mechanisms underlying schizophrenia pathogenesis rather than neurobiological alterations indicative of the same causal mechanism.

In the longitudinal arm of our study, we aimed to test whether a pharmacological reduction in TSPO levels using natalizumab was associated with changes in ACC glutamate and glx levels. It has previously been shown that natalizumab treatment is associated with increases in lesional white matter glutamate in multiple sclerosis [57]. Despite this, we observed no significant differences in ACC glutamate or glx levels after natalizumab treatment, either within those who received treatment alone or when compared to placebo. We primarily hypothesised that natalizumab would cause changes in glutamate and glx levels by reducing microglial activation. The present negative finding may therefore be due to the fact that natalizumab also failed to induce changes in TSPO DVR [31]. Alternatively, given the lack of a cross-sectional relationship between TSPO DVR and ACC glutamate and glx levels, the lack of effect of natalizumab may be due to a lack of a relationship between TSPO expression and glutamate dysregulation.

Since there was a reduction in total, positive and negative PANSS in the natalizumab group, we performed an exploratory analysis testing for an association between change in ACC glutamate and glx levels and change in symptom measures, but no significant association was observed. Previous studies have reported reductions in ACC and medial frontal cortex glutamate levels after antipsychotic treatment [21, 58, 59], although evidence of an association with symptom reduction is mixed [59]. Our findings suggests that the reduction in symptoms observed in the treatment group occurred independently of changes in ACC glutamate or glx levels.

This study’s strengths included a large cohort of individuals with first-episode psychosis and controls, significantly larger than most PET TSPO and MRS glutamate studies in schizophrenia [12, 60]. While it is therefore possible that negative findings are due to type II error, this study was powered to identify small-moderate effects size (f^2^ = 0.08 in the cross-sectional analysis and Cohen’s d = 0.72 in the longitudinal analysis). Smaller relationships are unlikely to be clinically significant. Second, the use of simultaneous PET and MR enables near simultaneous measurement of TSPO binding and ACC metabolite concentrations, avoiding a time gap or scanner bias. Third, we showed in sensitivity analyses that our findings were robust to potential confounders including age, sex, TSPO binding status and current smoking status (Supplementary Tables 2–5 and 9). Finally, we used a second generation TSPO-PET tracer, which has approximately twice the binding affinity for TSPO than first generation tracers for medium and high affinity binders [61].

A limitation to our study was that we performed ^1^H-MRS in the ACC and not in other brain regions. We focused on the ACC due to evidence linking ACC glutamate and glx levels to symptom severity and inadequate response to antipsychotic medication [21, 24]. Nevertheless, it is possible that altered glutamate or glx levels in other brain regions may be associated with TSPO expression. Future studies should consider performing ^1^H-MRS in multiple regions, in particular the thalamus and basal ganglia which show the most robust differences in glutamate levels in individuals with schizophrenia compared to healthy controls [60]. Secondly, we used a mixed RCT and open-label study design for the longitudinal arm of the study due to disruption caused to the study by the coronavirus pandemic. Pooling data from these two arms of the study may have introduced bias and heterogeneity to the analysis, although it is unlikely that our main outcomes were influenced by expectation and the procedures were identical across the two arms, besides blinding. There was also no change in our findings when analysing the two groups separately. We also used 3 T MRS with a PRESS sequence to quantify glutamate and glx. Glutamate and glutamine MRS spectra peaks overlap when using 3 T and are more difficult to discriminate at lower echo times. Although the PRESS sequence constrains echo times to 30 ms, it is still difficult to reliably differentiate glutamate from glutamine. We therefore reported glx in addition to glutamate. Furthermore, use of a single MRS voxel inevitably samples across subregions of the ACC, which is structurally and functionally heterogenous [62]. Given its proximity to the genu, our voxel predominantly sampled the pregenual and dorsal ACC. Nevertheless, the size of the voxel means that not all subregions are likely to be sampled and there may be some inter-individual differences in the regions samples. Future studies can address this limitation by employing smaller voxels and multi-voxel approaches [63]. Another consideration is the use of a pseudo-reference region to calculate DVR. Nevertheless, there were no difference in tracer uptake within the pseudo-reference region between patients and controls, nor any effect of natalizumab treatment on the pseudo-reference region [31]. It has also previously been shown that non-specific binding of TSPO tracer is equivalent in individuals with schizophrenia and healthy controls [64], supporting the validity of TSPO DVR calculated using reference region approaches. Our sample included individuals receiving stable doses of antipsychotic medication, predominantly second-generation antipsychotics such as aripiprazole and olanzapine. Antipsychotics may influence both glutamate levels and neuroinflammation. However, this cannot explain the null findings compared to controls and antipsychotic use was broadly similar across treatment groups. Finally, we included an unstratified cohort of individuals with first-episode psychosis, which may incorporate multiple different aetiological subgroups. Future clinical studies investigating these hypotheses may therefore benefit from enriching their samples for individuals with prior evidence of disturbed immune activation or glutamatergic dysregulation, respectively.

In conclusion, we found no evidence of an association between TSPO binding and ACC glutamate or glx levels in healthy individuals or with first-episode psychosis. These findings do not support the hypothesis that microglial dysfunction drives ACC glutamate dysregulation in first-episode psychosis. Notwithstanding the possibility of untested regional relationships and the study’s methodological limitations, our findings may indicate that ACC aberrant microglia activity and glutamatergic dysregulation represent two distinct mechanisms contributing to the neurobiology of schizophrenia.

## Supplementary information

Supplementary information

CONSORT Checklist

## Data Availability

Drs Hindley and Marques had full access to all the data used in the study and take responsibility for its integrity and the accuracy of data analysis. The dataset generated during the present study is not publicly available to preserve participant confidentiality, but can be made available based on a data-sharing agreement through the corresponding author.
