# Supplementary material for: Does a monoclonal antibody targeting immune cells affect glutamatergic level in schizophrenia? A multimodal PET/MRS brain imaging study
Source: Neuropsychopharmacology. 2026 Jul 9;51(11):2083–92. doi: 10.1038/s41386-026-02466-3 (PMC13597488; doi:10.1038/s41386-026-02466-3)
Supplement: Supplementary file 2 [file 41386_2026_2466_MOESM1_ESM.docx]

Does a monoclonal antibody targeting immune cells affect glutamatergic level in schizophrenia? A multimodal PET/MRS brain imaging study

Guy Hindley^1,2,3^*, Yuya Mizuno^3,4,5^, Katherine Beck^3,6^, Ines Carreira Figueiredo^3^, Toby Pillinger^3,6^, Sami Jeljeli^7^, Joel Dunn^7^, Alexander Hammers^7^, Ramla Awais^8^, Kerstin Sander^8^, Erik Årstad^8^, David J Lythgoe^9^, Julia J Schubert^9^, Mattia Veronese^9,10^, Federico Turkheimer^93^, Oliver D Howes^3,6^, Tiago Reis Marques^3†^

^1^ Centre for Precision Psychiatry, Institute of Clinical Medicine, University of Oslo and Division of Mental Health and Addiction, Oslo University Hospital, 0407 Oslo, Norway

^2^ Department of Psychiatry, Lovisenberg Diaconal Hospital, Oslo, Norway

^3^ Department of Psychosis Studies, Institute of Psychiatry, Psychology and Neurosciences, King’s College London, 16 De Crespigny Park, London SE5 8AB, United Kingdom

^4^ East London NHS Foundation Trust, London, United Kingdom

^5^ Department of Neuropsychiatry, Keio University School of Medicine, Tokyo, Japan

^6^ South London and Maudsley NHS Foundation Trust, London, United Kingdom

^7^ School of Biomedical Engineering and Imaging Sciences, King's College London, United Kingdom

^8^ Centre for Radiopharmaceutical Chemistry, University College London, United Kingdom

^9^ Department of Neuroimaging, King’s College London, United Kingdom

^10^ Department of information Engineering, University of Padua, Italy

* Joint last author

† Corresponding author

Correspondence:	Guy Hindley

Centre for Precision Psychiatry

Institute of Clinical Medicine

University of Oslo, 0407 Oslo

Norway

[g.f.l.hindley@medisin.uio.no](mailto:g.f.l.hindley@medisin.uio.no)

Running title:	Glutamate and microglia activity in schizophrenia

Supplementary figure 1: Example of MRS voxel placement for the ACC and voxel segmentation into grey matter (GM), white matter (WM), and cerebrospinal fluid (CSF) using SPM 12 and Gannet 3.1 to enable correction of the metabolite concentration.

Supplementary figure 2:Example of MRS spectrum. The raw data is plotted in black, the fit in red, and the residuals at the top of the plot.

Supplementary figure 3: MRI structural images (A & D), PET images (summed 10–60 min post-injection, B&D), and regions of interest (C&F) for an example healthy control (A-C) and first episode psychosis patient (D-F).

Supplementary figure 4: Illustrating the construction of the pseudo-reference region in a patient with first episode psychosis (A) and a healthy control (B). Red squares indicate GM voxels exhibiting kinetic behaviour most consistent with minimal specific binding from which the pseudo-reference region was calculated using a supervised clustering algorithm.

A

Supplementary figure 5: Comparing standardised uptake values (SUV) of the pseudo-reference region pre (v1) and post (v2) treatment with placebo or natalizumab in individuals with first episode psychosis (n=48). There was no significant difference when tested using a two-tailed paired Student’s t test.

B

Supplementary figure 6: Scatterplots illustrating the relationship between ACC metabolite concentrations (x-axis) and symptom severity as quantified using the Positive and Negative Syndrome Scale (PANSS), Scale for the Assessment of Negative Symptoms (SANS), and the Calgary Depression in Schizophrenia Scale (CDSS). Lines of best fit and standard errors are estimated using linear models.

**Supplementary Figure 7.** CONSORT flow diagram summarizing study recruitment

^*1^ Nine patients who participated in the beginning of the study did not have PET imaging (four patients formed the sentinel group and had no imaging, five patients only had MRI); ^*2^ Of the 65 patients receiving baseline PET-MR scans, two were excluded from the MRS analysis and three were excluded from the PET analysis due to data quality issues; ^*3^ One patient randomised to placebo withdrew consent for intervention after the 1^st^ infusion, but completed follow-up assessments; ^*4^ Four patients receiving open-label natalizumab withdrew consent for intervention, but completed follow-up assessments (three patients after the 1^st^ infusion, one patient after the 2^nd^ infusion); ^*5^ Two participants who were excluded from the longitudinal PET analysis due to data quality issues underwent follow up MRI alone.

Supplementary figure 8: Box plots comparing ACC concentrations between patients (blue) and controls (red) for: glutamate (glu) and glutamate + glutamine (glx); and ACC TSPO DVR. Y axis = grey-matter corrected metabolite concentrations in the ACC (ratio of metabolite resonance area to the unsuppressed water resonance area) for MRS measures and grey matter TSPO DVR in the ACC region for PET measures.

Supplementary Table 1: Voxel Segmentation and Spectral Quality in the ACC

| **Parameter** | **HCs/**  **Mean (SD)** | **FEP/**  **Mean (SD)** | **FEP – follow-up/**  **Mean (SD)** | **Test statistic¨**  1) HC vs FEP  2) Baseline vs follow-up (paired) | **p-value** |
| --- | --- | --- | --- | --- | --- |
| Cramer-Rao Lower Bound  (Glu) | 5.82 (0.501) | 5.94 (0.689) | 6.49 (3.14) | Mann Whitney U = 1453.5  Mann Whitney U = 111 | 0.473  0.2308 |
| Cramer-Rao Lower Bound  (Glx) | 6.35 (0.622) | 6.46 (0.937) | 6.45 (0.673) | Mann Whitney U = 1423.5  Mann Whitney U = 180 | 0.660  0.622 |
| Full Width at Half Maximum | 0.0406 (0.00698) | 0.0396 (0.00698) | 0.409 (0.0168) | Mann Whitney U = 1269.5  Mann Whitney U = 368.5 | 0.556  0.821 |
| Signal-to-Noise Ratio | 36.5 (2.64) | 36.4 (3.18) | 36.9 (3.76) | t = 0.264  t = 0.079 | 0.792  0.937 |
| Gray Matter, % | 66.7 (5.70) | 64.5 (5.70) | 64.9 (4.79) | t = 1.89  t = 0.285 | 0.0619  0.777 |
| White Matter, % | 15.6 (4.76) | 16.7 (3.79) | 16.6 (4.07) | t = -1.31  t = 0.497 | 0.193  0.621 |
| Cerebrospinal Fluid, % | 18.7 (3.81) | 18.8 (4.70) | 18.4 (4.01) | t = -1.22  t = -0.913 | 0.224  0.366 |

Glu - glutamate corrected; Glx - glutamate + glutamine corrected.

Supplementary Table 2: Results for Sensitivity analysis 1 - Testing for an association between ACC TSPO DVR and ACC glutamate (glu) and glutamate + glutamine (glx) concentrations using linear regression, controlling for TSPO genotype status only

| ACC metabolite | Sum | Patients (n=61) | Controls (n=39) |
| --- | --- | --- | --- |
| Glu | β=-0.004, SE=0.006, p=0.490 | β=-0.009, SE=0.008, p=0.244 | β=-0.005, SE=0.008, p=0.529 |
| Glx | β=-0.004, SE=0.004, p=0.308 | β=-0.005, SE=0.005, p=0.334 | β=-0.003, SE=0.006, p=0.618 |

Supplementary Table 3: Results for Sensitivity analysis 2 - Testing for an association between ACC TSPO DVR and ACC glutamate (glu) and glutamate + glutamine (glx) concentrations using linear regression, controlling for age, sex and TSPO genotype status.

| ACC metabolite | Whole sample (n=100) | Patients (n=61) | Controls (n=39) |
| --- | --- | --- | --- |
| Glu | β=-0.003, SE=0.006, p=0.553 | β=-0.009, SE=0.008, p=0.244 | β=0.001, SE=0.009, p=0.865 |
| Glx | β=-0.004, SE=0.004, p=0.263 | β=-0.008, SE=0.005, p=0.117 | β=-0.001, SE=0.006, p=0.908 |

Supplementary Table 4: Results for Sensitivity analysis 3 - Testing for an association between ACC TSPO DVR and ACC glutamate (glu) and glutamate + glutamine (glx) concentrations using linear regression among high affinity binders only

| ACC metabolite | Whole sample (n=100) | Patients (n=61) | Controls (n=39) |
| --- | --- | --- | --- |
| Glu | β=0.006, SE=0.007, p=0.342 | β=0.0002, SE=0.009, p=0.987 | β=0.007, SE=0.010, p=0.485 |
| Glx | β=0.001, SE=0.004, p=0.779 | β=-0.004, SE=0.006, p=0.512 | β=0.005, SE=0.007, p=0.452 |

Supplementary Table 5: Results for Sensitivity analysis 4 - Testing for an association between ACC TSPO DVR and ACC glutamate (glu) and glutamate + glutamine (glx) concentrations using linear regression when controlling for current smoking status.

| ACC metabolite | Whole sample (n=100) | Patients (n=61) | Controls (n=39) |
| --- | --- | --- | --- |
| Glu | β=-0.002, SE=0.006, p=0.709 | β=-0.003, SE=0.008, p=0.670 | β=-0.002, SE=0.010, p=0.822 |
| Glx | β=-0.001, SE=0.004, p=0.724 | β=-0.002, SE=0.005, p=0.644 | β=-0.001, SE=0.007, p=0.881 |

Supplementary Table 6: Testing for an association between total grey matter TSPO DVR and ACC glutamate (glu) and glutamate + glutamine (glx) concentrations using linear regression when controlling for TSPO genotype status, sex and age.

| ACC metabolite | Whole sample (n=100) | Patients (n=61) | Controls (n=39) |
| --- | --- | --- | --- |
| Glu | β=-0.001, SE=0.002, p=0.787 | β=-0.0002, SE=0.003, p=0.955 | β=0.0005, SE=0.004, p=0.895 |
| Glx | β=-0.001, SE=0.002, p=0.555 | β=-0.0005, SE=0.002, p=0.817 | β=-0.0003, SE=0.003, p=0.918 |

Supplementary Table 7: Testing for an association between frontal lobe grey matter TSPO DVR and ACC glutamate (glu) and glutamate + glutamine (glx) concentrations using linear regression when controlling for TSPO genotype status, sex and age.

| ACC metabolite | Whole sample (n=100) | Patients (n=61) | Controls (n=39) |
| --- | --- | --- | --- |
| Glu | β=0.003, SE=0.005, p=0.549 | β=-0.002, SE=0.006, p=0.792 | β=0.012, SE=0.07, p=0.120 |
| Glx | β=-0.0005, SE=0.003, p=0.860 | β=-0.001, SE=0.004, p=0.712 | β=0.002, SE=0.006, p=0.789 |

Supplementary Table 8: Testing for an association between temporal lobe grey matter TSPO DVR and ACC glutamate (glu) and glutamate + glutamine (glx) concentrations using linear regression when controlling for TSPO genotype status, sex and age.

| ACC metabolite | Whole sample (n=100) | Patients (n=61) | Controls (n=39) |
| --- | --- | --- | --- |
| Glu | β=0.0005, SE=0.004, p=0.903 | β=0.003, SE=0.005, p=0.477 | β=-0.002, SE=0.006, p=0.695 |
| Glx | β=-0.001, SE=0.003, p=0.712 | β=0.001, SE=0.003, p=0.828 | β=-0.002, SE=0.004, p=0.628 |

Supplementary Table 9: Testing for a change in ACC glutamate (Glu) and glutamate + glutamine (Glx) after treatment with natalizumab with **time** (pre-treatment vs. post-treatment) as the main fixed effect, controlling for age, sex and current smoking status using ANCOVA.

| ACC metabolite | Variable | Sum square | F value | P-value |
| --- | --- | --- | --- | --- |
| Glu | Time  Current smoking status  Age  Sex | 0.822  0.173  0.886  0.053 | 1.21  0.254  1.30  0.0778 | 0.277  0.616  0.259  0.781 |
| Glx | Time  Current smoking status  Age  Sex | 1.40  0.001  0.552  2.16 | 1.06  0.0008  0.418  1.64 | 0.307  0.978  0.521  0.206 |

Supplementary Table 10: Testing for an association between change in ACC glutamate and glutamate + glutamine (glx) and percentage change in PANSS total scores, PANSS positive scores and PANSS negative scores in individuals treated with natalizumab after controlling for age and sex using linear regression

| ACC metabolite | PANSS total scores (n=32) | PANSS positive scores (n=32) | PANSS negative scores (n=32) |
| --- | --- | --- | --- |
| Glu | β=-3.09, SE=9.03, p=0.735 | β=-0.0580, SE=6.78, p=1.00 | β=-8.93, SE=9.10, p=1.00 |
| Glx | β=-16.7, SE=11.0, p=0.414 | β=-4.74, SE=8.49, p=1.00 | β=--4.01 SE=11.6, p=1.00 |

Supplementary Table 11: Testing for an association between change in ACC glutamate and glutamate + glutamine (glx) and percentage change in PANSS total scores, PANSS positive scores and PANSS negative scores in individuals treated with three doses of natalizumab

| ACC metabolite | PANSS total scores (n=32) | PANSS positive scores (n=32) | PANSS negative scores (n=32) |
| --- | --- | --- | --- |
| Glu | β=-3.09, SE=9.03, p=0.735 | β=-0.0580, SE=6.78, p=1.00 | β=-8.93, SE=9.10, p=1.00 |
| Glx | β=-16.7, SE=11.0, p=0.414 | β=-4.74, SE=8.49, p=1.00 | β=--4.01 SE=11.6, p=1.00 |

# Supplementary Methods

## Randomisation and masking

During the first phase of the study, patients with first-episode psychosis were randomised 1:1 in blocks of four to receive once-monthly infusions of natalizumab 300mg or placebo (i.e., NaCl 0.9% saline infusions of matching volume). Computer randomisation was carried out centrally at the King’s Clinical Trials Unit, and both participants and investigators (including raters) were blinded to allocation. Independent pharmacists and research nurses, who were unblinded, were responsible for preparing natalizumab 300mg or saline solutions.

## Concomitant care

Participants received treatment as usual from Early Intervention in Psychosis teams alongside the intervention, including ongoing medical treatment with a stable dose of any antipsychotic medication besides clozapine for a minimum of one month before screening.

# Supplementary Results

## Participant exclusions

Four first episode psychosis patients (FEP) were recruited as part of the sentinel group and had no imaging. Two FEP were excluded from the MRS analysis due to failing the quality control procedure. Seven additional FEP were excluded from the PET analysis due to abnormal PET degradation dynamics (n=1), limited datapoints after PET acquisition (n=1), and no PET data having been acquired due to delayed delivery of the PET tracer (n=5). Two healthy controls (HC) were excluded from the MRS analysis due to no MRS data having been acquired. One additional HC was excluded from the PET analysis due to low contrast of the PET data.

## Baseline association between ACC metabolite concentrations and symptom severity

There were no significant associations between ACC metabolite concentrations and symptom severity at baseline as quantified using the PANSS, SANS or CDSS (All p > 0.05, Supplementary Figure 2).
